# Imprecisão ameaçadora: superando vieses no conceito de cuidado paliativo

**DOI:** 10.1590/0102-311XPT063425

**Published:** 2025-07-04

**Authors:** Arthur Fernandes da Silva, Marileise Roberta Antoneli Fonseca, Aline Camera Cintra

**Affiliations:** 1 Secretaria de Estado de Saúde do Distrito Federal, Brasília, Brasil.; 2 Hospital de Clínicas da Universidade Estadual de Campinas, Campinas, Brasil.; 3 Hospital Santa Izabel da Santa Casa de Misericórdia da Bahia, Salvador, Brasil.

Prezadas Editoras,

Recentemente, tivemos a oportunidade de ler o artigo intitulado *Situações Difíceis e Sentimentos no Cuidado Paliativo Oncológico* ^1^, em que o coletivo autoral analisou o complexo tema das reações emocionais de profissionais no contexto do cuidado paliativo. Contudo, pelo menos duas expressões reverberaram de forma diferente da esperada para a Academia Nacional de Cuidados Paliativos.

Em primeiro lugar, a definição de cuidado paliativo como “(...) *uma abordagem destinada às pessoas com* doenças fatais*, de qualquer idade,* sem perspectiva curativa*, buscando qualidade no* tempo restante da vida” ^1^ (p. 2, grifos nossos) carece de ancoragem na respeitável produção acadêmica sobre o tema, ainda que identificada citação à literatura ^2^. Seja pelas definições oficiais e adequadamente interpretadas da Organização Mundial da Saúde (OMS) ^3^ ^,^ ^4^ ou da Associação Internacional de Hospices e Cuidados Paliativos (IAHPC, acrônimo em inglês) ^5^, o cuidado paliativo é propagado de forma ampla, como uma abordagem ou campo de saberes que estão comprometidos com o alívio do sofrimento de pessoas e familiares convivendo com doenças graves ou ameaçadoras à vida e que prejudicam sua qualidade, atuando de forma holística em busca da prevenção e do alívio do sofrimento.

Nesse contexto, é possível justificar parte da compreensão popular e superficial de que cuidado paliativo se destina exclusivamente a pessoas em fim de vida ou, como habitualmente dito, “doentes terminais”. Também é possível e necessário estender nossa compreensão aos impactos dos vieses implícitos ^6^ (ou preconceitos inconscientes) sobre a população em geral, incluindo profissionais de saúde, com seus efeitos deletérios na assistência.

Contudo, cumpre apontar o desvio conceitual supracitado como potencial indutor de erros aos leitores, posto que reforça a visão estereotipada de cuidado paliativo como assistência possível diante da falta de “*perspectiva curativa*”, o que não é o caso. Resta posto na literatura e na prática em saúde, no mundo e no Brasil, que o cuidado paliativo se destina a todas as pessoas com quaisquer condições graves ou ameaçadoras ao longo de todos os ciclos de vida, sendo potencialmente melhor quanto mais precocemente for incorporado ao plano de cuidado do indivíduo e de sua família ^7^. Essa afirmação repousa serena sobre uma determinação da 67ª Assembleia Mundial de Saúde da OMS ^7^, que demandou aos países-membros o fortalecimento do cuidado paliativo como componente de cuidados integrais nas Redes de Atenção à Saúde ao longo do curso vital, recomendando sua integração e oferta precoce na atenção primária à saúde.

Não obstante, a ênfase na expressão “*tempo restante da vida*” na citação também pode ser interpretada de forma limitante, uma vez que o cuidado paliativo não se baseia apenas na contagem do tempo que resta ao paciente, mas, sim, na busca contínua por qualidade de vida, por meio de uma abordagem holística que envolve controle da dor, sintomas, suporte emocional, psicológico e social, e, ainda, o acompanhamento da família. A integração de cuidados paliativos em momentos mais iniciais da doença ^8^ pode, inclusive, melhorar o prognóstico global do paciente ^9^, evitando hospitalizações frequentes e proporcionando uma vida mais ativa e satisfatória ^10^.

Portanto, a citação falha ao reduzir o cuidado paliativo a um momento específico da trajetória da doença, sem refletir a complexidade e a pluralidade dos benefícios dessa abordagem, que é essencialmente centrada no paciente, na sua qualidade de vida e no seu bem-estar, independentemente da expectativa de vida restante.

A Política Nacional de Cuidados Paliativos, instituída pela *Portaria nº 3.694* do Gabinete da Ministra da Saúde em 22 de maio de 2024, formaliza a demanda por um esforço coletivo de agentes públicos e sociedade civil organizada para a promoção de uma cultura de cuidado paliativo, envolvendo discussões sobre vida, viver, morte e morrer ^11^. Isso posto, é dever coletivo a propagação de conceitos cientificamente corretos e eticamente inquestionáveis, cuidadosamente pensados para construir pontes entre a academia e a sociedade, os profissionais de saúde e o público leigo, os formuladores, executantes e os afetados pela implementação de políticas públicas.

Dessa forma, ressaltamos a importância de utilizar definições conceitualmente sólidas e alinhadas com o conhecimento científico atualizado sobre cuidados paliativos. A forma como esses conceitos são apresentados em publicações acadêmicas e científicas influencia diretamente na percepção de profissionais da saúde, de gestores e da sociedade em geral sobre a abordagem paliativa. É essencial evitar reduções conceituais que possam restringir o acesso precoce aos cuidados paliativos e perpetuar estereótipos que limitam seu impacto positivo. Agradecemos a oportunidade de dialogar sobre esse tema e reforçamos nosso compromisso com a disseminação de informações claras e embasadas, que contribuam para o aprimoramento da assistência às pessoas que vivem com doenças graves e suas famílias.
